# Comparative evaluation of chlorhexidine gluconate with alcohol and polyhexamethylene biguanide with Tris-EDTA as antiseptic solutions for pre-operative skin preparation in dogs

**DOI:** 10.14202/vetworld.2024.2451-2459

**Published:** 2024-11-05

**Authors:** Nithida Boonwittaya, Chompoonek Yurayart, Pareeya Udomkusonsri, Saroch Kaewmanee, Somchai Sompaisarnsilp, Naris Thengchaisri, Taksaon Duangurai

**Affiliations:** 1 Department of Companion Animal Clinical Sciences, Graduate Student in Veterinary Clinical Studies, Faculty of Veterinary Medicine, Kasetsart University, Bangkok 10900, Thailand; 2 Department of Microbiology and Immunology, Faculty of Veterinary Medicine, Kasetsart University, Bangkok 10900, Thailand; 3 Department of Pharmacology, Faculty of Veterinary Medicine, Kasetsart University, Bangkok 10900, Thailand; 4 Department of Animal Science, Faculty of Agriculture, Kasetsart University, Bangkok 10900, Thailand; 5 Faculty of Veterinary Medicine, Rajamangala University of Technology Tawan-Ok, Chonburi 20110, Thailand; 6 Department of Companion Animal Clinical Sciences, Faculty of Veterinary Medicine, Kasetsart University, Bangkok 10900, Thailand

**Keywords:** alcohol, antisepsis, chlorhexidine gluconate, dogs, polyhexamethylene biguanide, skin preparation, tris-ethylenediaminetetraacetic acid

## Abstract

**Background and Aim:**

Skin antisepsis plays a crucial role in pre-operative skin preparation, with chlorhexidine gluconate and alcohol being historically the preferred choice. However, concerns have risen regarding the development of bacterial resistance to chlorhexidine. Polyhexamethylene biguanide (PHMB) combined with Tris-ethylenediaminetetraacetic acid (Tris-EDTA) has recently emerged as a skin and wound antiseptic. This study aimed to compare the antibacterial efficacy and local safety of 2% chlorhexidine gluconate with 70% alcohol (CG+Alc) and 0.3% PHMB with 6% Tris and 1.86% EDTA (PHMB+Tris-EDTA) for pre-operative skin preparation in dogs.

**Materials and Methods:**

Twenty-four adult dogs underwent aseptic preparation on both sides of their ventral abdomens, with one side receiving CG+Alc and the other side receiving PHMB+Tris-EDTA, assigned randomly. Skin swab samples were collected pre-antisepsis and at 3-, 10-, and 60-min post-antisepsis to quantify bacterial colony-forming units (CFUs). Local skin reactions (erythema and edema) were evaluated after hair clipping, pre-antisepsis, and at 3-, 10-, 30-, and 60-min post-antisepsis.

**Results:**

There was no significant difference in bacterial CFU counts between the two antiseptic groups pre-antiseptic. Both solutions significantly reduced CFU counts (p < 0.05) at all post-antisepsis sampling times compared with pre-antisepsis. However, dogs treated with PHMB+Tris-EDTA showed a significantly higher incidence of edema at 10 min (p = 0.02) and 30 min (p = 0.003) and a higher incidence of erythema at 10 min (p = 0.043) post-antisepsis compared with CG+Alc. No skin reactions were observed in either group at 60 min post-antisepsis.

**Conclusion:**

CG+Alc and PHMB+Tris-EDTA reduced bacterial counts in pre-operative skin preparation in dogs. However, acute transient skin reactions were observed more frequently following the application of PHMB+Tris-EDTA.

## Introduction

Preventing surgical site infections (SSIs) is critically important in clinical practice. SSIs can increase morbidity and mortality, systemic infections, potentially fatal outcomes, and higher treatment costs [1–3]. Using antibiotics alone during surgery is insufficient because pathogens often exhibit antibiotic resistance. The overuse of antibiotics can lead to adverse effects and increased resistance [4, 5]. Effective decontamination and asepsis of surgical sites are essential steps to reduce bacterial load, eliminate pathogenic microorganisms, minimize the introduction of opportunistic pathogens into surgical wounds, and decrease the risk of SSI [6, 7]. Antiseptics are used to achieve these goals. An ideal antiseptic should have broad-spectrum efficacy, rapidly and sustainably eliminate pathogens, not induce resistance, and exhibit low local toxicity and systemic adverse effects [8, 9]. Despite the availability of various antiseptics for skin preparation, comparative studies on their efficacy are limited [10–17]. Most studies emphasize the importance of preventing SSI, whereas methods to improve the efficiency of reducing bacterial load before surgery remain limited.

Chlorhexidine has been widely used for pre-operative skin preparation because of its broad-spectrum antimicrobial activity and prolonged residual effect [6]. A previous study by Hibbard *et al*. [18] has shown that chlorhexidine in alcohol significantly reduces bacterial counts and provides prolonged antimicrobial action compared with chlorhexidine or alcohol alone. One meta-analysis confirmed the superiority of chlorhexidine over alcohol, particularly in clean-contaminated surgical procedures. The 2% chlorhexidine in 70% alcohol solution is widely used. However, increasing the SSI concentration beyond 2% did not significantly reduce SSI risk and was linked to adverse effects, such as allergic reactions [19]. Chlorhexidine-alcohol is significantly more effective than povidone-iodine in preventing both superficial and deep incisional SSIs [19, 20]. The rapid onset of action of alcohol, due to its ability to coagulate and denature cytoplasmic membrane proteins, resulting in cell lysis and disruption of cell metabolism, combined with the long residual effect of chlorhexidine, yields a synergistic effect [6, 21]. However, the rise of antibiotic-resistant bacteria raises concerns about potential resistance to long-used antiseptics like chlorhexidine. Some bacteria exhibit resistance to chlorhexidine and cross-resistance to antibiotics [6, 22, 23]. In addition, chlorhexidine can cause local skin reactions, including contact dermatitis, immediate and delayed hypersensitivity, and increased pain when applied to wound areas [6, 24]. Given the limitations of chlorhexidine, there has been growing interest in alternative antiseptic agents such as polyhexamethylene biguanide (PHMB). PHMB is structurally similar to naturally occurring antimicrobial peptides, which disrupt bacterial cell walls, leading to cell death without resistance. PHMB effectively reduces bacterial load, pain, and biofilm formation [25]. Ethylenediaminetetraacetic acid (EDTA) can bind and enhance the permeability of bacterial cell membranes while disrupting biofilm structures by capturing essential metals, rendering it an effective tool for controlling and eliminating biofilm formation. The combined use of EDTA and antiseptics enhances the penetration of antiseptics through the bacterial cell walls and exerts synergistic effects in reducing bacterial load [26].

This study aimed to compare the antibacterial efficacy and local safety of 2% chlorhexidine gluconate with 70% alcohol (CG+Alc) and 0.3% PHMB with 6% Tris and 1.86% EDTA (PHMB+Tris-EDTA) for pre-operative skin preparation in dogs. By evaluating these specific combinations, we hope to provide valuable insights into their relative effectiveness and safety, which may help guide clinical decision-making in pre-operative skin preparation protocols.

## Materials and Methods

### Ethical approval and Informed consent

This study was approved by the Kasetsart University Institutional Animal Care and Use Committee (approval ID #ACKU66-VET-004) and by the Ethics Review Board of the Office of National Research Council of Thailand (license U1-09430-2564). Written consent was obtained from all dog owners. The experiment complied with the Kasetsart University Institutional Animal Care and Use Standards.

### Study period and location

The study was conducted from February 2023 to February 2024 at Kasetsart University Veterinary Teaching Hospital, Bangkok, Thailand.

### Animals

Twenty-four healthy, client-owned adult dogs (six males and 18 females) with a median age of 28.5 months (range: 7–104 months) and a median weight of 19.73 kg (range: 11.7–33.25 kg) were enrolled in this study. All dogs were fully vaccinated, had no visible skin conditions, and had no history of current disease or injury. They underwent a thorough physical examination, and their hematological and basic serum chemistry results were within normal limits. Water and food were withheld for 4 and 6 h before general anesthesia was administered.

### Anesthesia

All dogs were anesthetized using the same protocol with a slow infusion of propofol (Troypofol 1% w/v, Troikaa Pharmaceuticals Ltd., Uttarakhand, India) at a dose of 2–6 mg/kg intravenously (IV), followed by premedication with midazolam (Midazolam-hameln 0.5%, Siam Bioscience Co. Ltd., Nonthaburi, Thailand) at a dose of 0.2 mg/kg IV. Anesthesia was then maintained using sevoflurane (SEVO 100%, Singapore Pharmawealth Lifesciences Inc., Laguna, Philippines) and oxygen. Ringer’s lactate (5 mL/kg/h IV) was continuously administered throughout the anesthetic procedures until the dog was extubated [27]. Neither antibiotics nor anti-inflammatory drugs were administered during the study period.

### Pre-antiseptic skin preparation

Hair clipping was performed from the caudoventral thoracic area to the pubic area and from the midline to the bilateral sides using sterilized electric clippers and sterilized blades. Gas plasma sterilization was used for the sterilization process. Any skin reactions were assessed and recorded after clipping. In male dogs, hair in the preputial area was clipped, and the area was cleaned according to standard surgical room procedures, similar to the vagina in female dogs. Draining the urine was performed with a urinary catheter, which was removed before transferring the dogs to the operating room.

Staff wearing surgical caps, masks, and gloves performed pre-antiseptic skin preparation using a non-antiseptic neutral detergent solution applied to sterile abdominal sponges, which were then wiped in a back-and-forth motion from the midline outward [28]. The area was rinsed with sterile water, and the process was repeated at least 3 times until no dust, hair, or soil remained. After drying with a sterile paper towel, the first culture sample was collected, and skin reactions were assessed. All dogs were examined in the same surgical room and monitored throughout the anesthetic period.

### Antiseptic skin preparation

The study area was divided from the ventral midline to each lateral edge of the prepared skin. Both sides of the prepared area were randomly assigned to antiseptic groups. One side received a solution of 2% chlorhexidine gluconate and 70% ethyl alcohol (Prepskin-c, Medicpharma Co. Ltd., Samut Sakhon Thailand), while the other received a solution of 0.3% PHMB and Tris-EDTA (6% Tris and 1.86% EDTA) (TEP-Plus; Panomix Nutrisolutions Co. Ltd., Patum Thani, Thailand). These antiseptic solutions, which were clear and colorless and had similar viscosities, were dispensed into separate sterile stainless-steel bowls prepared by non-investigator staff members. The investigator was blind to the specific antiseptic at the end of the procedure. Staff wearing surgical caps, masks, and sterile surgical gloves performed skin antiseptic application using a sterile syringe to slowly rinse off the solution droplets and thoroughly cover the assigned skin area until saturated. After 3 min, the excess solution was carefully removed using dry sterile paper towels. Surgical drapes were then placed on all four sides to cover hairy skin using a standard technique, and a second culture sample was obtained through skin reaction assessment. The third and fourth culture samples were collected at 10 and 60 min, with continued evaluation of skin reactions at 10, 30, and 60 min after antiseptic application.

### Bacterial sample collection

**Figure-1:**
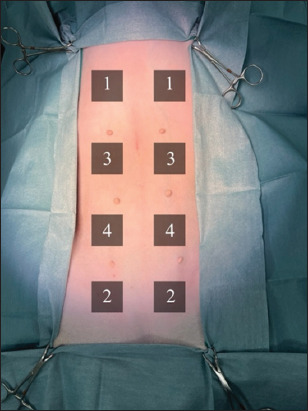
Locations of the sample collection sites on both sides. Each site was numbered according to the collection sequence.

Skin bacteria samples were collected from four designated skin regions on both sides: the cranial 1/4^th^ (for the first sample), caudal 1/4^th^ (the second sample), cranial 2/4^th^ (the third sample), and caudal 2/4^th^ (the fourth sample). Each region was numbered from 1^st^ to 4^th^ based on the collection sequence (Figure-1). A modified swabbing method was performed using a sterile cotton swab (Sterile cotton swab, Thai Gauze Co. Ltd., Bangkok, Thailand) across a 4 × 4 cm square area within a sterilized acrylic frame at the specified skin location. Each swab was placed in a collecting tube containing 5 mL of buffered peptone water. All samples were immediately transported to the laboratory, centrifuged, and diluted to a 10-fold serial dilution from 10 to 10^−3^. A volume of 100 μL of each diluted sample was collected and spread onto blood and MacConkey agar. The plates were placed in an incubator at 35 ± 2°C for 48 h. After incubation, bacterial colonies were counted and recorded. A reincubation for an additional 48–72 h was performed for plates with few or no bacterial colonies and bacterial count was conducted again [13, 29, 30]. Results were reported as colony-forming units per milliliter (CFU/mL), calculated based on the dilution series.

### Evaluation of adverse skin reactions to antiseptic solutions in dogs

Adverse skin reactions were assessed by a single investigator who remained consistent throughout the study and was blinded to the type of antiseptics used on each skin site. The assessment was performed after hair clipping, pre-antiseptic wash, and 3-, 10-, 30-, and 60-min post-antisepsis. Adverse skin reaction was identified and classified as “reaction” if any evidence of erythema and/or edema formation was observed, and “no reaction” if no such reaction was observed. The scoring criteria were adapted from the OECD Guideline [31], with skin reaction scores categorized as follows: No reaction = 0, Slight (mild redness; well-defined swelling <1 mm) = 1, Moderate (moderate redness; 1 mm swelling) = 2, and Severe (eschar formation; above 1 mm swelling or expanding beyond the application area) = 3.

### Statistical analysis

The sample size was calculated using G*Power software version 3.1.9.7 (https://www.psychologie.hhu.de/arbeitsgruppen/allgemeine-psychologie-und-arbeitspsychologie/gpower) to detect differences in CFU counts between pre-antiseptic and post-antiseptic sampling times with a power of 80% and an alpha error of 0.05. Shapiro–Wilk test was used to assess data normality, and the results were indicated using non-parametric methods for analysis. Bacterial counts at each sampling time point were reported as median and interquartile range (IQR). The Mann–Whitney U test was used for pair-wise comparisons of bacterial counts between antiseptic groups at each sampling time. The Wilcoxon signed-rank test was used to compare bacterial counts within antiseptic groups at different post-antiseptic sampling times compared with the pre-antiseptic (first) sampling time. Comparisons of adverse skin reactions in the PHMB+Tris-EDTA group were performed using the presence of skin reactions in the CG+Alc group as a reference at each sampling time. Results were reported as odds ratios and were analyzed using Fisher’s exact test. The statistical analysis was performed using R Statistical Software version 4.3.2 (https://www.r-project.org/). Statistical significance was set at p < 0.05.

## Results

**Figure-2:**
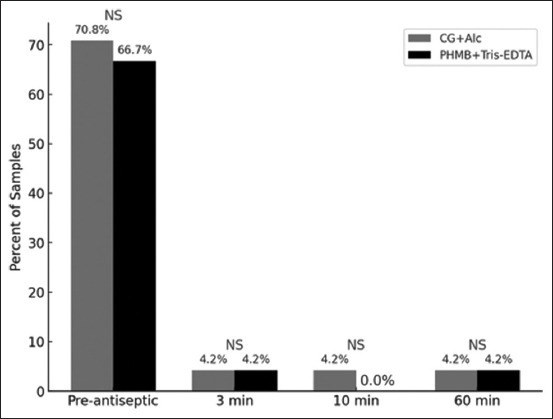
Percentage of samples with positive bacterial results in the chlorhexidine gluconate with alcohol group and polyhexamethylene biguanide with Tris-ethylenediaminetetraacetic acid group. There was no statistically significant difference (NS) comparing between two groups at different time point.

In this study, 48 samples were collected at each sampling time (24 samples per group). The percentage of samples with positive bacterial growth is presented in Figure-2. When comparing the proportion of positive samples in the PHMB+Tris-EDTA group with those in the CG+Alc group, no significant differences were observed at any time point.

The median and IQR of bacterial counts (CFU/mL/swab) in each group at different sampling times are presented in Table-1. In the matched-paired comparisons, the bacterial counts were significantly lower after antiseptic application at the second, third, and fourth sampling times compared with the pre-antiseptic sampling times for both the CG+Alc (p = 0.0003, p = 0.0005, p = 0.0003) and PHMB+Tris-EDTA (p = 0.0005, p = 0.0005, p = 0.0005) groups. Similarly, there were no significant differences in bacterial counts between both groups at the initial (p = 1.000), second (p = 1.000), third (p = 0.338), and fourth (p = 1.000) sampling times.

**Table-1:** Median and IQR of bacterial counts (CFU/mL/swab) at different time points and paired comparisons within each antiseptic group.

Antiseptics	Pre- antiseptics	3 min	10 min	60 min
CG+Alc				
Median	87.5	0.0	0.0	0.0
IQR	0.0–206.2	0.0–0.0	0.0–0.0	0.0–0.0
Range	0–2125	0–25	0–25	0–50
p-value		0.0003*	0.0005*	0.0003*
PHMB +Tris-EDTA				
Median	62.5	0.0	0.0	0.0
IQR	0.0–250.0	0.0–0.0	0.0–0.0	0.0–0.0
Range	0–5200	0–25	0-0	0–100
p-value		0.0005*	0.0005*	0.0005*

* = p *<* 0.05 considered statistically significant. IQR=Interquartile range, CG + Alc=Chlorhexidine gluconate with alcohol, PHMB + Tris-EDTA=Polyhexamethylene biguanide with Tris-ethylenediaminetetraacetic acid

This study recorded adverse skin reactions in 48 areas per evaluation time. Four of the 24 dogs exhibited adverse skin reactions after hair clipping. Two dogs showed level 2 erythema, and the other two showed level 1 erythema. All four dogs continued to exhibit adverse skin reactions at the same level after cleaning with a neutral detergent solution (pre-antiseptic time). There was no evidence of edema formation in these dogs. The remaining 20 dogs did not show any adverse skin reactions throughout the pre-antiseptic process, and they were subsequently used to evaluate the evidence of skin reactions after the antiseptic application.

A total of 8/20 dogs (37.5%) presented with adverse skin reactions after antiseptic treatment in the PHMB+Tris-EDTA group. Of these, five dogs exhibited reactions during the 3–30-min evaluation period, while three dogs showed adverse effects during the 10–30-min period. Only 1 dog (5%) experienced adverse skin reactions in the CG+Alc group, which occurred during the 3–10-min period. The severity of adverse skin reactions for each case is presented in Table-2.

**Table-2:** Adverse skin reaction levels after using antiseptics in dogs with normal skin reaction following the pre-antiseptic process.

Antiseptics	No.	Erythema formation				Edema formation			
	
		3 min	10 min	30 min	60 min	3 min	10 min	30 min	60 min
CG + Alc	1	1	1	0	0	1	1	0	0
PHMB + Tris-EDTA	1	1	1	1	0	1	1	1	0
	2	1	1	0	0	1	2	1	0
	3	1	1	0	0	1	2	1	0
	4	1	1	0	0	1	2	1	0
	5	2	2	1	0	2	3	2	0
	6	0	1	0	0	0	1	1	0
	7	0	2	1	0	0	2	1	0
	8	0	0	0	0	0	1	1	0

CG + Alc=Chlorhexidine gluconate with alcohol, PHMB + Tris-EDTA=Polyhexamethylene biguanide with Tris-ethylenediaminetetraacetic acid

When comparing the risk of presenting adverse reactions as classified as “reaction” after using antiseptics in 20 dogs with absence of skin reactions after pre-antiseptic process, the skin area treated with PHMB+Tris-EDTA had a 10.2 times higher risk of developing erythema at 10 min compared with CG+Alc antiseptic (p = 0.043), and a 12.7 times higher risk of developing edema at the same time compared with CG+Alc antiseptic (p = 0.020). Furthermore, at 30 min, no edema formation was observed in the skin of dogs treated with CG+Alc antiseptic. In contrast, the skin prepared with PHMB+Tris-EDTA had a significantly higher risk of edema formation compared with CG+Alc (p = 0.003), as shown in Tables-3 and 4.

**Table-3:** Occurrence of erythema formation after antiseptic use in dogs with normal skin reactions following the pre-antiseptic process.

Antiseptics	Number of dogs with erythema formation (n = 20)			

	3 min (%)	10 min (%)	30 min (%)	60 min (%)
CG + Alc				
No reaction	19 (95)	19 (95)	20 (100)	20 (100)
Reaction	1 (5)	1 (5)	0 (0)	0 (0)
PHMB + Tris-EDTA				
No reaction	15 (75)	13 (65)	17 (85)	20 (100)
Reaction	5 (25)	7 (35)	3 (15)	0 (0)
p-value	0.182	0.043*	0.231	

* p < 0.05 considered statistically significant. CG + Alc=Chlorhexidine gluconate with alcohol, PHMB + Tris-EDTA=Polyhexamethylene biguanide with Tris-ethylenediaminetetraacetic acid

**Table-4:** Occurrence of edema formation after antiseptic use in dogs with normal skin reactions following the pre-antiseptic process.

Antiseptics	Number of dogs with edema formation (n = 20)			

	3 min (%)	10 min (%)	30 min (%)	60 min (%)
CG + Alc				
No reaction	19 (95)	19 (95)	20 (100)	20 (100)
Reaction	1 (5)	1 (5)	0 (0)	0 (0)
PHMB + Tris-EDTA				
No reaction	15 (75)	12 (60)	12 (60)	20 (100)
Reaction	5 (25)	8 (40)	8 (40)	0 (0)
p-value	0.182	0.020*	0.003*	

* p *<* 0.05 considered statistically significant. CG + Alc=Chlorhexidine gluconate with alcohol, PHMB + Tris-EDTA=Polyhexamethylene biguanide with Tris-ethylenediaminetetraacetic acid

## Discussion

This study demonstrated that both antiseptic solutions effectively reduced bacterial counts, with no significant difference in the bacterial load between the two groups at various time points. In this study, the pre-operative skin preparation included hair clipping and cleaning the skin with a neutral detergent lacking demonstrated antimicrobial activity before applying the antiseptics. The electric clippers and blades were sterilized before use to reduce cross-contamination between dogs, as previously reported by Boucher *et al*. [12], Melekwe *et al*. [13], and Asimus *et al*. [17]. Razor blades were not used because they increase the risk of bacterial growth on injured skin [3, 32]. Hair clipping after anesthesia induction also reduces the risk of injury to conscious dogs and decreases the incidence of SSIs when performed within 4 h before preoperative skin cleaning [33].

Regarding the skin cleaning process, some studies have used an antiseptic solution since the initial wash to remove dirt and debris [15, 17], and some have used an antiseptic-based scrub or a combination of antiseptics and skin cleaners to remove dirt and eliminate bacteria in a single step [13, 16, 17]. However, this study separated the steps by first removing surface contaminants with a neutral detergent lacking antimicrobial properties, followed by the application of antiseptics as the final step. This approach aimed to eliminate the mechanical factors of scrubbing that reduce bacterial counts from the efficacy of antiseptics and to prevent issues with the reduced efficacy of most antiseptics on dirty skin with organic residues, in accordance with previous studies and veterinary recommendations [10, 12, 13, 33, 34].

The swabbing method was used to collect bacterial samples from the dogs’ skin in this study, which is a common technique for sampling the skin microbiome in both humans and dogs [35–40], providing bacterial profiles similar to those of skin biopsy [35]. This method was used in previous studies by Belo *et al*. [11] and Melekwe *et al*. [13] to compare the efficacy of antiseptics on canine skin. Using sterile cotton swabs reduces contamination and maintains the aseptic technique. The same brand and size of swabs were used, and the same person collected the samples throughout the study to minimize variability in bacterial counts due to pressure, swabbing direction, type, and swab size [41]. Furthermore, the swab test method allows accurate bacterial enumeration when high bacterial loads are present, especially in the first sample, because it can be diluted before culture and CFUs can be calculated.

Regarding the sampling sites, specific areas of 4 × 4 cm were designated, which were large enough to collect samples without overlap in 10 locations for dogs weighing 10 kg or more to prevent the effects of repeated sampling on bacterial counts and antiseptic solutions in subsequent sampling rounds. While other studies collected samples from sites related to surgery and at risk of infection [10, 12] or from the same sites without specifying separate locations [11, 13, 15, 16], the sampling methods varied among the studies. Therefore, future research should investigate and establish appropriate guidelines for bacterial sampling methods for comparing the efficacy of antiseptics.

Each antiseptic solution was directly dropped onto the skin on each side to avoid the effects of mechanical factors, such as scrubbing or rubbing, on bacterial counts, skin irritation, and the transfer of bacteria from the initial site to other areas. In addition, spraying techniques were avoided to compare two antiseptic solutions simultaneously on the same dog to prevent cross-contamination between the two sides, which could impact the evaluation of antiseptic efficacy. Furthermore, a single application of antiseptic solutions following skin cleaning with a non-antiseptic neutral detergent in this study significantly reduced bacterial counts, further increasing confidence in the efficacy of the solutions when used in clinical settings where multiple applications are common. Although some studies collected samples immediately after the application of antiseptics [11, 13, 17], we allowed for a specified contact time to ensure a more accurate evaluation of their effectiveness. This study employed a contact time of 3 min before sample collection, which is consistent with many clinical studies on dogs [10, 12, 15, 16, 42]. The bactericidal efficacy of chlorhexidine is time-dependent in preoperative skin preparation, with initial bacterial counts decreasing as the contact time increases. Several studies recommend leaving chlorhexidine on the skin for at least 2–3 min before surgery, but when combined with alcohol, the contact time can be reduced to 30 s [6, 19, 28, 43, 44]. However, no clinical studies have investigated the optimal contact time before surgery in PHMB+Tris EDTA. In the consensus on wound antiseptic: update 2018, data suggested that a 3-min contact time with PHMB solution effectively prevented SSI in contaminated traumatic wounds in humans [9]. Available PHMB products have a relatively wide concentration range, from 0.1% to 20%, resulting in varying bacterial reduction times in wounds [45]. The results of sample collection 3 min after the application of the antiseptics showed a median bacterial count of 0 CFU/mL/swab, indicating that both solutions significantly reduced bacterial counts within this timeframe.

Furthermore, this study extended the sample collection times to 10 and 60 min, covering the durations of most basic surgical procedures, to compare the efficacy of bacterial reduction at fixed time points and evaluate the residual effect over the surgical period. This differs from some studies in which samples were collected after surgery at varying times for each case [10, 12] or only immediately before and after the application of the antiseptics [11]. It was also observed that in the third (10 min) and fourth (60 min) sample collections, the number of dogs with detectable bacteria did not increase from the second collection. The median value remained at 0 CFU/mL/swab, which differs from many studies that found an increase in the number of dogs with bacteria and bacterial counts over time following the application of antiseptics [10, 12, 15, 16, 42]. This preliminary investigation can be extended to studies with longer sample collection times, more complex surgical procedures, and varying skin qualities in dogs.

The results showed no significant difference in bacterial CFUs between the two groups at any sampling time point, and both antiseptic solutions significantly reduced skin bacterial load compared with pre-antisepsis levels. These findings suggest that PHMB+Tris-EDTA is a viable alternative to the widely used CG+Alc for decontaminating dog skin. The combination of PHMB and Tris-EDTA offers several potential advantages. PHMB has a broad spectrum of antimicrobial activity and a unique mechanism of action involving the disruption of bacterial cell membranes [25]. This difference in mechanism may reduce the likelihood of cross-resistance between PHMB and antibiotics, a concern that has been raised with chlorhexidine [6, 22, 23]. The addition of Tris-EDTA enhances the antimicrobial efficacy of PHMB by chelating divalent cations in the bacterial cell wall, increasing membrane permeability, and facilitating the penetration of PHMB [26]. This synergistic effect may allow the use of lower concentrations of PHMB, potentially reducing the risk of adverse effects. However, PHMB+Tris EDTA has not been studied as a pre-operative skin antiseptic, and there is no information about their immediate and residual effects. A consensus on wound antiseptic mentioned PHMB in the context of wound irrigation or wound dressing for contaminated or infected wounds in human medicine [9]. In veterinary medicine, PHMB solution is used to irrigate bite wounds in dogs, significantly reducing bacterial counts observed when the contact time exceeds 15 min. However, the volume of solution used was not standardized and was left to the discretion of the surgeon [46]. Another study by Boucher *et al*. [12] comparing pre-operative skin preparation antiseptics in dogs found that CG+Alc was more effective in reducing bacterial counts than a combination of quaternary ammonium compounds, PHMB, and alcohol. Note that the PHMB concentration used in this study was only 0.05%.

This study reported local skin reactions after hair clipping, unlike most other antiseptic trials did not report. Notably, 17% of the dogs exhibited erythema without any accompanying swelling. Dogs with pre-existing skin problems before hair clipping were excluded to prevent the effect of lesions on the evaluation of adverse skin reactions to antiseptics. The number of dogs or severity of adverse reactions did not increase after skin cleaning with a neutral detergent, which differs from previous studies by Lambrechts *et al*. [10] and Boucher *et al*. [12]. This may be attributed to the fact that the cleaning process was performed by experienced researchers and team members who regularly work in the operating room and thoroughly understand the research principles and procedures, thus reducing the risk of injury from scrubbing during the initial cleaning process. Therefore, only the results from 20 dogs with normal skin after hair clipping and initial skin cleaning were analyzed to assess adverse reactions from antiseptic exposure.

Despite the similar efficacy in reducing skin bacterial counts, the PHMB+Tris-EDTA group, which used a 0.3% PHMB solution combined with Tris-EDTA, had a significantly higher incidence of transient adverse skin reactions, particularly erythema and edema, in the short period after application. Typically, PHMB is an antiseptic with a lower incidence of allergic reactions than other agents [47]. Nonetheless, a dose-response relationship for local adverse skin reactions cannot be excluded from the study. Combining PHMB and its added additives can enhance antimicrobial efficacy and cytotoxicity. Although most PHMB products are formulated with macrogol or betaine, which are generally better tolerated by tissues than other antiseptics, it is important to recognize that these and other additives can significantly influence PHMB’s overall antimicrobial and safety profile [47, 48]. The products used in this study also contain Tris-EDTA. Compared with other studies using Tris-EDTA and topical antiseptics in dogs, no clinical adverse reactions were observed [49, 50]. However, this could be because these studies were conducted in dogs with pre-existing otitis externa and skin lesions, making it challenging to detect additional adverse skin reactions caused by the agents. Furthermore, the concentration of Tris-EDTA was considerably lower in these studies. One study using wipes impregnated with a mixture of antibiotics, antiseptics, and Tris-EDTA to clean canine skin did not report any adverse reactions, but the concentration of Tris-EDTA was not clearly specified [51].

## Conclusion

This study demonstrated that 0.3% PHMB with 6% Tris and 1.86% EDTA might be an effective alternative to 2% CG+Alc for preoperative skin antiseptic in dogs, with similar efficacy in reducing skin bacterial counts. However, acute transient skin reactions were observed more frequently in the PHMB+Tris-EDTA group than in the CG+Alc group. This study compared PHMB+Tris-EDTA and CG+Alc but excluded other commonly used antiseptic agents, limiting a comprehensive understanding of their relative effectiveness and safety. Adverse reactions were observed and recorded only up to 60 min after application, potentially missing delayed effects or long-term outcomes. Variability in the incidence and severity of skin reactions among dogs may affect the reliability of the findings. Conducted at a single center, the results may have limited applicability to other settings or populations. In addition, the lack of objective assessments of skin reactions could limit the accuracy and introduce bias in the evaluation outcome. Addressing these limitations will help confirm and extend these findings, offering a more comprehensive understanding of the comparative effectiveness and safety of antiseptic agents. Future studies should involve larger sample sizes, a broader range of surgical procedures, more diverse patient populations, longer follow-up periods, and objective assessments of skin reactions.

## Authors’ Contributions

NB, CY, PU, NT, and TD: Conceived and designed the study. NB, CY, and TD: Conducted comprehensive literature search, performed the study, and collected data. NB, CY, SK, and SS: Analyzed the data. NB, SK, and TD: Drafted the manuscript and prepared the tables and figures. CY, PU, SK, NT, and TD: Critically reviewed and modified the manuscript. NB, CY, SK, and TD: Performed final manuscript revision. All authors have read and approved the final manuscript.
